# Twisted Rubber Variable-Stiffness Artificial Muscles

**DOI:** 10.1089/soro.2018.0129

**Published:** 2020-06-02

**Authors:** Tim Helps, Majid Taghavi, Sihan Wang, Jonathan Rossiter

**Affiliations:** ^1^ Department of Engineering Mathematics, University of Bristol, Bristol, United Kingdom.; ^2^ Bristol Robotics Laboratory, Bristol, United Kingdom.

**Keywords:** twisted, rubber, artificial, muscle, variable-stiffness

## Abstract

Variable-stiffness artificial muscles are important in many applications including running and hopping robots, human–robot interaction, and active suspension systems. Previously used technologies include pneumatic muscles, layer and granular jamming, series elastic actuators, and shape memory polymers. All these are limited in terms of cost, complexity, the need for fluid power supplies, or controllability. In this article, we present a new concept for variable-stiffness artificial muscles (the twisted rubber artificial muscle, TRAM) made from twisted rubber cord that overcomes these limitations. Rubber cord is inexpensive, readily available, and inherently compliant. When an extended piece of rubber cord is twisted, the tensile force it exerts is reduced and its stiffness is altered. This behavior makes twisted rubber ideal for use as an artificial muscle, because its output force and natural stiffness are both controllable by varying twist angle. We investigate the behavior of four types of rubber cord and evaluate which type of rubber allows for the greatest reversible reduction in average stiffness (fluoroelastomer [FKM standard] rubber, 56.42% reduction) and initial stiffness (silicone rubber, 92.62%). Tensile force and stiffness can be further altered by increasing the twist angle of the artificial muscle beyond a threshold angle, which initiates nonlinear buckling behavior. This, however, can cause plastic deformation of the artificial muscle. Using a single TRAM, we show how the equilibrium position and natural frequency of a system can be simultaneously altered by controlling twist angle. We further demonstrate independent position and stiffness control of a functional robotic arm system using an antagonistic pair of TRAMs. TRAMs are ready for immediate inclusion in a wide range of robotic systems.

## Introduction

Variable-stiffness materials, whose compliance can be controllably altered, are useful in multiple engineering fields from robotics to transportation. Compliance can be beneficial to running and hopping systems, allowing energy to be stored and reused as the leg shortens and extends,^1^ and variation in leg stiffness can allow a runner to alter their stride frequency.^2^ In human–robot interaction, variable-stiffness systems can be used to optimally trade safety and performance.^3^ In the automotive industry, variable-stiffness suspension systems can considerably improve ride comfort, stability, and handling.^4^ Variable-stiffness materials have also been proposed for wearable orthotics to address movement impairments such as foot drop.^5^

Various physical phenomena and technologies have been proposed to deliver materials with controllable stiffness. In laminar or layer jamming, interleaved thin sheets form a variable-stiffness structure. The sheets are free to slide past one another, allowing extension and flexion, until a vacuum is applied to the device, drawing the sheets together and preventing deformation.^6–11^ Similarly, in granular jamming, a particulate media such as coffee grounds contained within an elastic bag is transitioned from a compliant and malleable state to a rigid state by application of a vacuum, which causes the granular material to lock or “jam” together.^12,13^ Other systems have used antagonistic arrangements and variable-reduction-ratio transmissions that allow traditional linear springs to deliver variable-stiffness behavior.^14–16^ Similarly, the Mechanically Adjustable Compliance and Controllable Equilibrium Position Actuator (MACCEPA) actuator combines a tension-adjustable traditional linear spring and an offset lever arm to deliver decoupled controllable stiffness and equilibrium position for rotary joints.^17,18^ Many polymers exhibit glass transition behavior, transitioning from a hard, brittle, “glassy” state to a soft, flexible, “rubbery” state when heated above their glass transition temperature. This behavior has been well studied as a mechanism to deliver controllable stiffness variation.^5,19–24^ More unusual variable-stiffness mechanisms include an elastic ring with anisotropic stiffness, which can be rotated to change its loading direction resulting in a change in effective stiffness,^25^ and flexible structures coated in or containing wax, which phase transitions to a liquid when heated.^26^

**FIG. 1.**
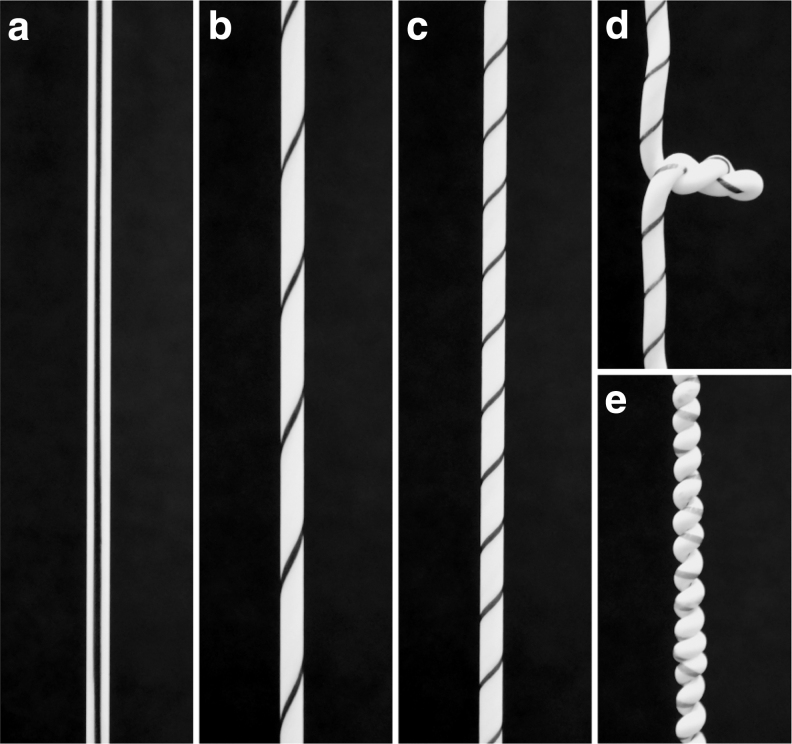
Twisted rubber. When rubber cord **(a)** is twisted, it can adopt a range of forms, including twisted **(b, c)**, buckled **(d),** and coiled **(e)**, depending on the twist angle and tensile load applied. In this article, we focus on the variable-stiffness behavior of the twisted form.

**FIG. 2.**
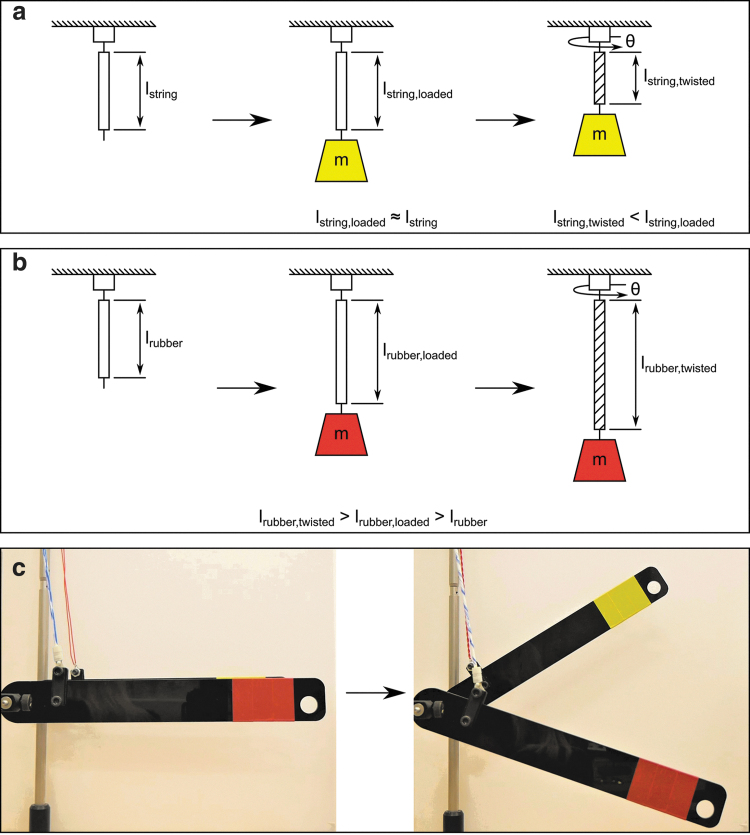
Comparison of a twisted string actuator with a TRAM. **(a)** Twisted string actuator: Adding a mass to the twisted string actuator negligibly effects its length, because the string has a very high stiffness, therefore *l*_string,loaded_ ≈ *l*_string_. Increasing the twist angle of the twisted string actuator results in it shortening, such that *l*_string,twisted_ < *l*_string,loaded_. **(b)** TRAM: Adding a mass to the TRAM increases its length, since it is elastic, therefore *l*_rubber,loaded_ > *l*_rubber_. Increasing the twist angle of the TRAM results in it lengthening further, such that *l*_rubber,twisted_ > *l*_rubber,loaded_. **(c)** Experimental comparison of twisted string actuator (*yellow lever arm*) and TRAM (*red lever arm*). The same number of twists and twist direction is applied to both actuators. The twisted string actuator shortens, raising the *yellow lever arm*, whereas the TRAM lengthens, lowering the *red lever arm*. TRAM, twisted rubber artificial muscle. Color images are available online.

In this article, we highlight the variable-stiffness behavior of twisted rubber (Fig. 1) and demonstrate its suitability for use as an artificial muscle. The twisted rubber artificial muscles (TRAMs) presented here take inspiration from twisted string actuators,^27–35^ whereby one or more strings are twisted to exert a linear contractile force. Here, we replace the comparatively inelastic strings in twisted string actuators with a single strand of rubber cord. Surprisingly, when a load is suspended from a rubber cord and the cord is twisted, the cord length *increases*, in contrast to the shortening-with-twist behavior exhibited by twisted string actuators (Fig. 2).

In the case of the twisted string actuator, twisting causes the actuator to assume a coiled (single string actuator) or helical (double string actuator) form, reducing its length (Fig. 2a). In contrast, increasing the twist angle of loaded TRAM results in it increasing in length (Fig. 2b). Although the TRAM's form is twisted (rather than buckled or coiled), more twisting further increases its length. In the same way as adding additional load to an elastic cord increases its extension, twisting applies forces and compressive stresses that squeeze the elastic cord and causes it to extend.

**FIG. 3.**
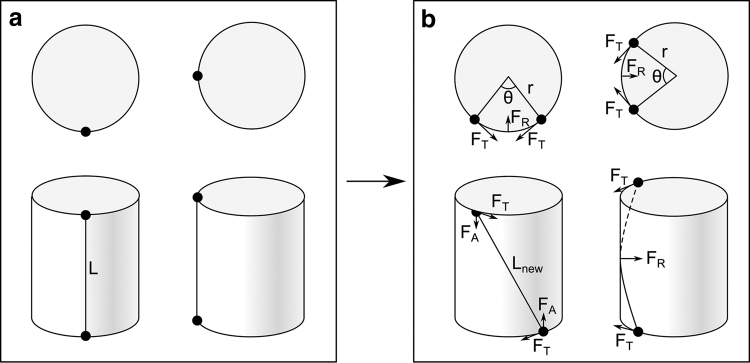
Cylinder analogy demonstrating inward radial force induced by twisting. **(a)** A single cord of elastic material on the surface of a rigid cylinder. **(b)** Rotating the ends of the cord away from one another by a twist angle θ stretches and displaces the cord, resulting in a distributed load on the surface of the cylinder that can be resolved into an inward radial force *F*_R_.

The source of the twist-induced compressive stress that acts to squeeze the rubber cord is not immediately clear; the mechanism can be imagined more clearly by considering an analogous system, a single cord of elastic material, lying vertically on the surface of a cylinder (Fig. 3a). If the ends of the elastic cord are rotated a twist angle θ away from one another, the cord is displaced and stretched by a length ΔL:

Eq. 1 $$\Delta L=\sqrt{L^{2}+r^{2}\theta^{2}}-L,$$

where *L* is the initial length of elastic cord and the height of the rigid cylinder and *r* is the radius of the rigid cylinder. Because the cord is elastic and has been stretched, it exerts a tensile force *T* resisting its extension. For a Hookean material,

Eq. 2 $$T=k\left(\sqrt{L^{2}+r^{2}\theta^{2}}-L\right),$$

where *k* is the material's stiffness. This force can be resolved into two components: axial force *F*_A_ and tangential force *F*_T_ (Fig. 3b), where

Eq. 3 $$F_{A}=T\sin\tan^{-1}\left(\frac{L}{r\theta }\right)$$

and

Eq. 4 $$F_{T}=T\cos\tan^{-1}\left(\frac{L}{r\theta }\right).$$

Because the elastic cord has been stretched around a curved surface, these forces induce a distributed load, which can be resolved into a resultant force *F*_R_ acting radially inwards on the cylinder.

Similarly, a twisted rubber cord can be imagined as an infinitesimal number of stretched and displaced elastic cord elements, each contributing a tangential, axial, and radial force. The contribution of elements will reduce depending on their proximity to the axis of the cylinder, with the central element contributing a negligible amount. The sum of the tangential forces *F*_T_ results in a torsion that resists the twist of the sample, and the sum of the radial forces *F*_R_ results in a compressive stress that acts to squeeze the cylinder. This squeezing will cause axial extension of the cylinder because of Poisson's effect, overcoming the sum of the axial forces *F*_A_ and resulting in an overall reduction in the tensile force. Furthermore, the compressive stress and extension applied to the rubber cord results in a change in its tensile stiffness because of the nonlinear stress–strain relationship of rubber. A similar change in stiffness could be achieved by prestretching the rubber cord in the axial direction; here application of twist allows tensile force and stiffness control without needing to control prestretch.

This phenomenon has been well studied in the field of materials.^36^ Using a Neo-Hookean stored energy function (SEF), the axial force reduction *F*_r_ is

Eq. 5 $$F_{r}=-\frac{\pi \psi^{2}r^{4}C_{10}}{2},$$

where *r* is the radius of the cylinder, *C*_10_ is the Neo-Hookean material constant and ψ is the torsion defined as φ/*l*, where φ is the total twist angle and *l* is the rubber cylinder length.^37^ Alternatively, using a Mooney SEF, the axial force reduction is

Eq. 6 $$F_{r}=-\pi \psi^{2}r^{4}\left(\frac{C_{10}}{2}+\frac{C_{01}}{\lambda }\right),$$

where *λ* is the stretch ratio along the axis of the cylinder, and *C*_10_ and *C*_01_ are the Mooney SEF coefficients.^37^ These models have been evaluated for twist angles up to 0.2 rad for a rubber sample of length 32 mm, implying a maximum ψ of 6.25 rad/m.^37^

Although the variation in axial tensile force exhibited by rubber cord when twisted has been studied in detail from a materials perspective,^36,37^ this phenomenon is yet to be investigated by the Soft Robotics community. Twisted rubber cord has extremely attractive qualities for use as artificial muscles: rubber cord is readily available, inexpensive, inherently compliant, and matches the typically desired artificial muscle form factor. It lacks the disadvantages associated with state-of-the-art artificial muscles such as delamination (layer separation) in dielectric elastomer actuator (DEA) stacks^38^ and does not require processing before use such as precoiling as in coiled polymer actuators.^39^ Twisted rubber cord can be included within a robotic system and its twist angle can be varied to control axial force. In addition, controlling the twist angle of rubber cord can deliver variable-stiffness behavior, which could improve the performance of locomotion robots or enhance safety in human–robot interaction.

## Materials and Methods

**FIG. 4.**
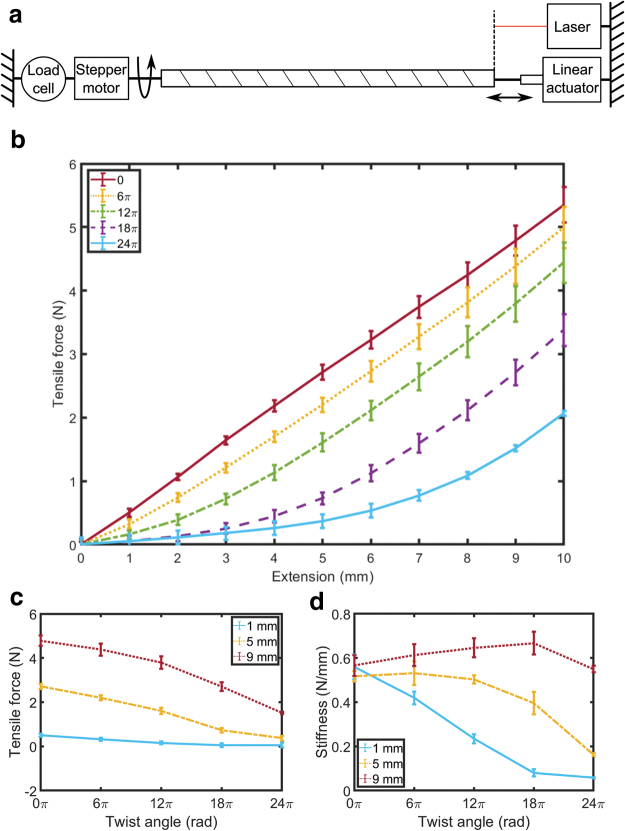
Materials testing setup and tensile behavior of 15 cm long, 3 mm diameter flouroelastomer (FKM standard) rubber cord. **(a)** A stepper motor controlled the twist angle of a rubber cord, and a linear actuator controlled its extension. The force exerted was recorded using a load cell, and extension was recorded using a laser displacement meter. **(b)** Force-extension behavior for different twist angles. Points are averages of lengthening and shortening data for three samples, and error bars show ±1 standard deviation. Each line shows data for one fixed twist angle from 0 to 24π. Force data for each line has been zeroed based on initial force; an example of unzeroed data is available in Supplementary Figure S1. Twisting the rubber cord alters its force-extension behavior, reducing output force. **(c)** Tensile force variation with twist angle, at different extensions. **(d)** Stiffness variation with twist angle, at different extensions. Stiffness was calculated using a forward difference first-order approximation. At low extensions, stiffness is reduced as twist angle is increased. At medium and high extensions, stiffness may increase or decrease as twist angle is increased. Color images are available online.

The force-extension behavior of rubber cord was investigated using a standard material testing setup (Fig. 4a). A NEMA-17 stepper motor (535-0489; RS, United Kingdom) driven by a stepper motor driver (HY-DIV268N-5A; Powlance, China) was attached to one end of a sample of rubber cord and used to control the twist angle. A linear actuator (LACT8P-12V-20; Pololu) was attached to the opposite end of the cord and was used to linearly extend and relax the cord. A load cell (DBCR-100N-002-000; Applied Measurements, United Kingdom) measured the tensile force exerted by the cord as it was extended and allowed to relax. A laser displacement meter (LK-G502; Keyence, Japan) recorded the movement of the linear actuator tip to infer the extension of the cord. Control signals were generated, and data were captured using a data acquisition device (NI USB-6229 BNC; National Instruments). During testing, the rubber cord was extended and relaxed by the linear actuator and tensile force was recorded throughout. Then, the stepper motor imparted a new twist angle and the rubber cord was once again extended and relaxed while tensile force was recorded. This process was repeated until the maximum twist angle was reached. Finally, the rubber cord was untwisted (its twist angle was returned to zero) and a final extension and relaxation was performed, and tensile force recorded to determine whether the untwisted stiffness of the sample had been altered (to confirm that plastic deformation had not occurred—if it had occurred then that trial's data were discarded). Because of entropic effects, rubber exhibits thermoelastic behavior, becoming warmer when stretched and cooler when relaxed. To ensure this did not influence results, the extension and relaxation of the sample were always performed slowly (maximum extension and relaxation speed 10 mm/s, with a 10 s delay between each extension–relaxation cycle) so that experiments involved a reversible, isothermal process.

## Results

### Tensile force and stiffness variation

Figure 4b shows force-extension behavior of a 15 cm long sample of the material that exhibited the greatest reduction in average stiffness: 3 mm diameter flueroelastomer (FKM standard) rubber cord. When the twist angle was zero (the sample is untwisted), force-extension behavior was roughly linear within this extension range. When the twist angle of the sample was increased, axial force at each displacement was reduced. Figure 4c shows tensile force reduction as twist angle was increased, with each line showing data at one value of extension; tensile force reduction was greatest when extension was large.

In addition to reducing tensile force, increasing twist angle alters the force-extension behavior of the rubber cord: the gradient of the force-extension curve changes, implying altered compliance. This is especially clear at low extensions: the gradient of the force-extension curve is considerably reduced, and the cord is considerably less stiff in the tensile direction. Figure 4d shows stiffness variation as twist angle was increased, again with each line showing data at one extension value. At low extension, stiffness was considerably reduced as twist angle was increased. In contrast, at medium and high extensions, the rubber cord's stiffness was increased as twist angle was increased to 6π and 18π, respectively, beyond which stiffness was reduced. We attribute this to the nonlinear stress–strain relationship of rubber, which is typically S-shaped. At small extensions, in the early region of the S-shaped stress–strain curve, the additional stress induced by twisting results in stiffness reduction. In contrast, at large extensions, in the latter region of the S-shaped stress–strain curve, the additional stress induced by twisting results in increase in stiffness.

### Maximum stiffness reduction

**FIG. 5.**
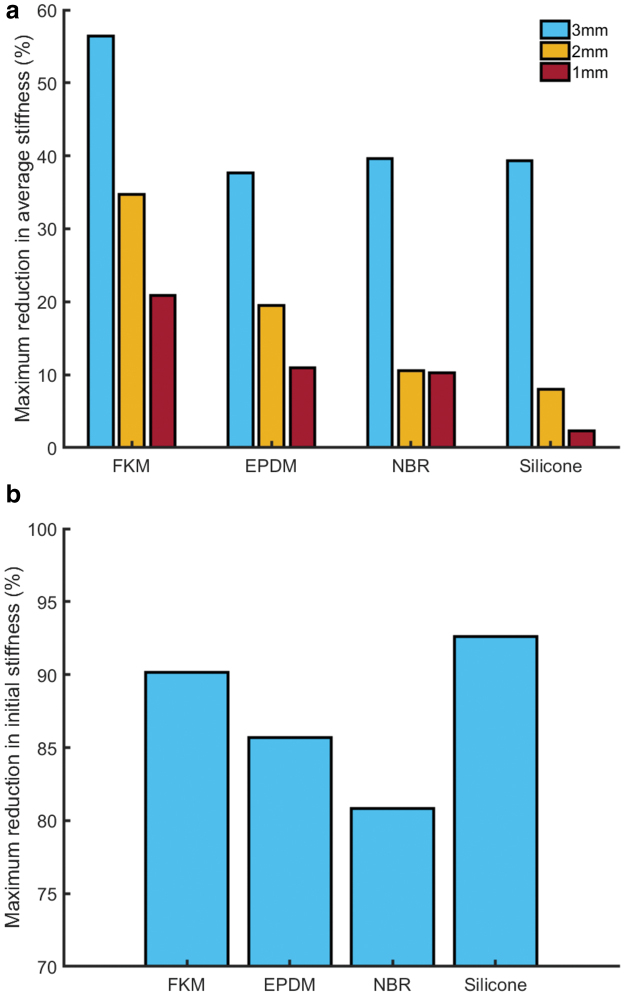
Maximum reversible reduction in average stiffness for different diameter and different material 15 cm long rubber cords, and maximum reversible reduction in initial stiffness for different material rubber cords of 3 mm diameter and 15 cm length. The materials that were tested were flouroelastomer (FKM standard) rubber, EPDM rubber, NBR rubber, and silicone rubber. **(a)** Average stiffness was calculated by dividing maximum tensile force by the maximum extension. Three millimeter diameter FKM rubber cord exhibited the greatest reduction (56.42%), undergoing a maximum extension of 7% and twist angle 24π. **(b)** Initial stiffness was calculated using a forward difference first-order approximation at 1 mm (0.6%) extension. For all materials, maximum twist angle was 24π. Silicone rubber cord exhibited the greatest reduction (92.62%). EPDM, ethylene propylene diene monomer; NBR, nitrile butadiene rubber. Color images are available online.

In general, for robotics applications, twisted rubber should be used within its elastic range, such that neither twisting or extension plastically deforms the artificial muscle. In cases where extension or torsion permanently altered the sample length or imparted some permanent twist (resulting in a slightly helical sample), these results were discarded. Results in Figure 5 thus show maximum *reversible* stiffness reduction (that which could be achieved without plastic deformation occurring).

For some robotics applications, particularly those where the entire extension range of an artificial muscle is required, an important metric is the amount by which overall stiffness of the muscle can be altered. We define this “average stiffness” as the mean stiffness over the full extension range of a rubber cord sample, calculated by dividing maximum tensile force by maximum reversible extension. Figure 5a shows the maximum reduction in average stiffness compared across different diameter and different material rubber cords: flouroelastomer (FKM standard) rubber, ethylene propylene diene monomer (EPDM) rubber, nitrile butadiene rubber (NBR), and silicone rubber. Depending on the material and diameter, different extensions and twist angles could be applied without permanently deforming the sample; the maximum applied extension was 12% and the maximum applied twist angle was 48π. Three millimeter diameter FKM rubber cord exhibited the greatest reduction in average stiffness (56.42%), undergoing a maximum extension of 7% and twist angle 24π, and is therefore a prime candidate material for a variable-stiffness artificial muscle.

For other applications, for example, where a system oscillates about its equilibrium position, only a small amount of extension will be applied to the artificial muscle. If a TRAM only experiences such small extensions, a much greater reduction in stiffness can be achieved. We define “initial stiffness” as the stiffness at an extension of 1 mm (0.6% of the length of the rubber cord). Figure 5b shows the maximum reduction in initial stiffness for different materials (all are 3 mm in diameter). In all cases, the maximum twist angle was 24π. All tested materials exhibited >80% reduction in initial stiffness, with twisted silicone rubber delivering the highest reduction of 92.62%.

### Buckling behavior

**FIG. 6.**
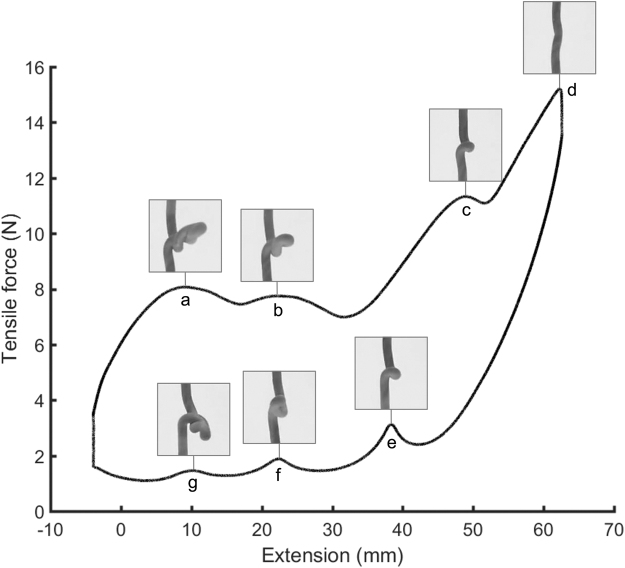
Force-extension behavior for a twisted sample of 3 mm diameter, 15 cm long, NBR rubber cord. The twist angle is 50π. As the cord is extended and relaxed, overall tensile force increases and decreases as each loop of the buckle is resolved and restored (points a, b and c for loading and points e, f and g for unloading). Maximum tensile force occurs at point d when all the buckle loops have been resolved and the cord is straight.

Thus far, analysis has been restricted to twist angles and extensions that do not permanently alter the rubber cord and cause plastic deformation. If this requirement is discarded, more complex behavior can be captured. At high twist angles, elastic rods can exhibit unusual buckling behavior, transitioning from a quasi-static short-wave helix form to a quasi-static writhing form.^40–42^ Figure 6 shows force-extension behavior for a 15 cm long sample of 3 mm diameter NBR rubber cord that has been twisted through 25 rotations (twist angle 50π). At this twist angle, the cord buckles outwards into a writhing form with three loops. As the cord is extended and relaxed, the overall tensile force increases and decreases as each loop of the buckle is resolved and restored (points a, b and c for loading and points e, f and g for unloading). Maximum tensile force occurs when all the buckle loops have been resolved and the cord is straight (point d). These deviations in tensile force could allow a single elastic element to have multiple controllable, stable extension points for a particular applied tensile load, which could be used in devices that require adjustable stable positions, such as medical gurneys and in robotic surgery. Alternatively, the large change in stiffness between a local minimum or maximum and a neighboring inflexion point could allow considerable controllable stiffness variation by only slightly altering extension or twist. However, because these large twist angles tend to cause plastic deformation and permanently alter the samples of rubber cord, untwisting the cord will not restore its original behavior—the artificial muscle should be used in this twist region only.

### Stiffness control

**FIG. 7.**
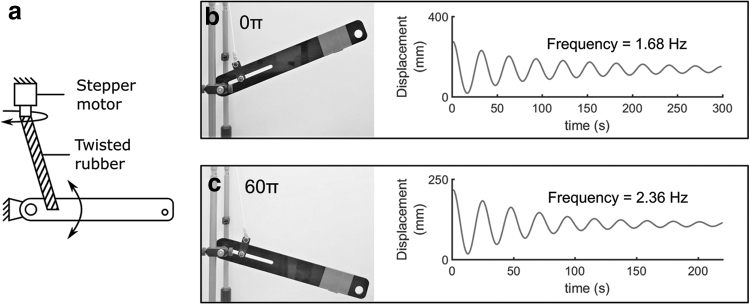
Comparison of natural frequency for arm supported by untwisted and twisted silicone rubber cord. **(a)** System diagram. **(b)** Arm supported by untwisted rubber cord; oscillation at a natural frequency of 1.68 Hz occurs when the arm is displaced and released. **(c)** Arm when the rubber cord twist angle is increased to 60π; oscillation now occurs at a natural frequency of 2.36 Hz when the arm is displaced and released, implying increased stiffness.

One application of TRAMs is controlling the natural frequency of a system. Figure 7 shows a simple robot arm supported by a single sample of silicone rubber cord. When the arm is displaced, and the cord is untwisted, it oscillates at a natural frequency of 1.68 Hz (Fig. 7a). When the twist angle of the rubber cord is increased, its stiffness, and correspondingly its natural frequency, is altered. Figure 7b shows the oscillation of the arm when the twist angle of the rubber cord is increased to 60π; the natural frequency has increased to 2.36 Hz. As can be seen from Figure 4d, at low extensions, increasing the twist angle of the rubber cord decrease the cord's stiffness; however, for large extensions, such as here where the weight of the arm has extended the rubber cord, increasing twist angle can increase stiffness. In a practical system the loading and twist angle should be monitored to determine the effective stiffness of the cord. This can be achieved by using a tensile force or strain sensor (for example, by coating with a strain-responsive resistive coating), potentially made from the same material as the rubber itself, and by monitoring the twist angle imposed by the motor. The ability of these artificial muscles to control the natural frequency of a system has wide-ranging applications, from improving the comfort and effectiveness in active suspension systems to increasing the efficiency and speed of legged locomotion robots.

Figure 7 shows how natural frequency of a system can be controlled and also demonstrates a shortcoming when a single TRAM is used—altering twist angle simultaneously alters both stiffness and equilibrium position. If stiffness needs to be altered, this can only be achieved by also altering equilibrium position. For a functional robotic system, stiffness and equilibrium position should be independently controllable. This functionality can be achieved using antagonistic arrangements of TRAMs.

### Independent stiffness and position control

**FIG. 8.**
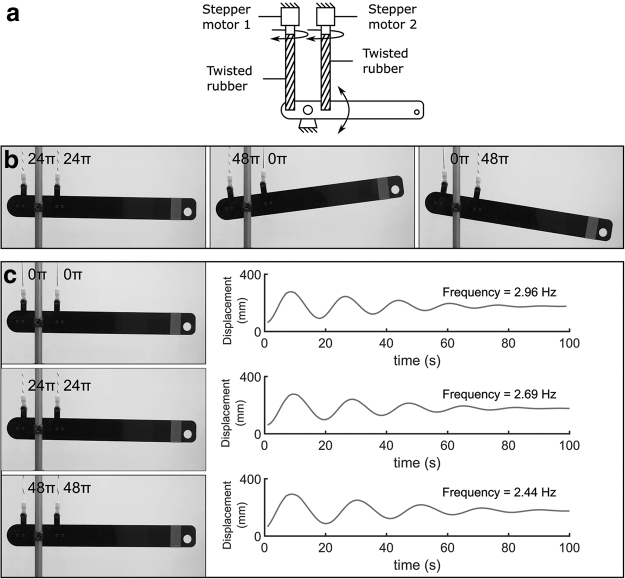
Controlling position and stiffness using an antagonistic pair of TRAMs. **(a)** System diagram. **(b)** Position control: controlling the twist angle of the *left* and *right* muscle relative to one another allows exertion of a clockwise or counterclockwise moment, controlling the arm's equilibrium angle between −8.28° and 8.43°. **(c)** Stiffness control: controlling the sum of the twist angle of the *left* and *right* muscle while keeping them equal allows the stiffness of the arm to be controlled without altering its rest angle.

Figure 8 shows a functional one-link robotic arm system driven by TRAMs. The muscles are configured in an antagonistic arrangement, which allows independent control of joint stiffness and equilibrium position. Joint stiffness can be increased by reducing the twist angle of both TRAMs, and because the force of both muscles is reduced equally by this twisting, equilibrium position is not affected. Alternatively, by adjusting the relative twist angle of the two TRAMs, equilibrium position may be controlled.

Figure 8b shows position control; when the twist angle of both TRAMs is equal (24π), the arm rests horizontally (0°). By increasing the twist angle of the left artificial muscle and reducing that of the right artificial muscle (to 48π and 0π, respectively), the tensile force exerted by the left muscle is reduced, and that of the right muscle is increased. This alters the arm's rest angle to −8.28°. Similarly, reducing the twist angle of the left muscle and increasing that of the right (0π and 48π respectively) moves the equilibrium position in the opposite direction, altering the arm's rest angle to 8.43°. These changes in angle are quite similar, highlighting the relatively high accuracy of TRAMs.

Figure 8c shows stiffness control. Because the twist angle of both TRAMs is equal, for an ideal system the arm should rest horizontally. If displaced, the arm oscillates at a natural frequency of 2.96 Hz. Increasing the twist angle of both TRAMs to 24π retains the horizontal equilibrium position but reduces stiffness and correspondingly reduces natural frequency to 2.69 Hz. Further increasing the twist angle of both TRAMs to 48π further reduces frequency to 2.44 Hz. The accuracy of this robotic system can be estimated by comparing how close the robot arm rests to its initial position in each different stiffness state. When the twist angle of both muscles was 24π, the deviation from initial position was 0.43°. When the twist angle of both muscles was 48π, the deviation from initial position was −0.28°. These values imply a standard deviation of 0.36° when the robot arm attempts to maintain a horizontal equilibrium position, confirming the high accuracy of the robotic system.

### TRAM applications

**FIG. 9.**
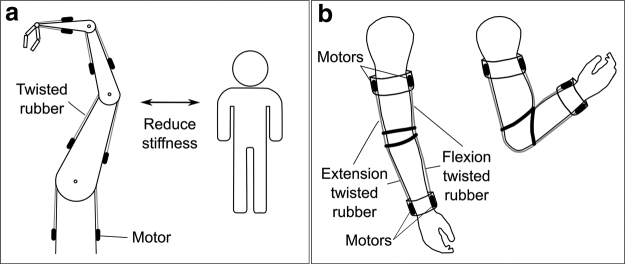
TRAM applications. **(a)** In human–robot interaction, the joint stiffness of a TRAM actuated robot arm could be reduced when the arm is approached by a human to improve safety, reducing the severity of harm in the case of a collision. **(b)** Wearable ART devices could control tensile force and stiffness for rehabilitation, muscle training, or power assist.

Figure 8 shows how antagonistic TRAMs can easily be used to independently control position and stiffness. This concept could allow for robots that are safer to interact with. Figure 9a shows a robot arm actuated entirely by TRAMs, with an antagonistic arrangement acting at each joint. This would allow the robot to reduce its joint stiffness when approached by a human, reducing the severity of harm caused in the case of a collision. A key application where TRAMs could be used is in assistive and rehabilitative technology (ART): wearable ART devices could be used for rehabilitation or muscle training, controlling the stiffness and force of joints to apply an appropriate amount of resistance (Fig. 9b). Alternatively, such devices could deliver assistive forces at the joints, imparting positive mechanical power to improve the mobility of people living with movement impairments.

## Conclusion

In this article, we introduced the new concept of TRAMs. These artificial muscles leverage the fundamental material behavior of twisted rubber, which exhibits a reduction in tensile force and change in stiffness when it is twisted. Twisting induces a compressive stress that reduces tensile force and lengthens the TRAM. The compressive stress also causes the stiffness of the TRAM to either increase or decrease (depending on loading) because of the nonlinear S-shaped stress–strain relationship of rubber. By controlling the twist angle of these artificial muscles, tensile force and stiffness can be controlled. Both average and initial stiffness can be reduced considerably (by up to 56.42% and 92.62%, respectively). This allows TRAMs to be used to control natural frequency of a system. Antagonistic arrangements of TRAMs further increase their utility, allowing for *independent* control of equilibrium position and stiffness, and enabling many applications in robotics and wearable assistive devices.

TRAMs do have limitations: compared with traditional (constant stiffness) engineering springs, which tend to store energy extremely efficiently; rubber has more associated viscous losses. This could increase energy requirements in, for example, running and hopping robots, and the benefits of controllable stiffness should be weighed against this reduced efficiency.

In future, we plan to test a wider range of elastic materials, to improve the range of available tensile force variation, stiffness control, and elastic range of TRAMs. We also plan to further investigate buckling and coiling behavior and how they may be exploited to deliver greater functionality in TRAMs. Finally, we plan to develop a proportional–integral–derivative (PID) controller that controls twist angle in a TRAM robotic system, characterize the performance of such a system, and demonstrate setpoint tracking and speed control.

## Data Access Statement

Data necessary to support conclusions are included in the article.

## Supplementary Material

Supplemental data
