# Supplementary material for: Twisted Rubber Variable-Stiffness Artificial Muscles
Source: Soft Robot. 2020 Jun 2;7(3):386–95. doi: 10.1089/soro.2018.0129 (PMC7301325; doi:10.1089/soro.2018.0129)
Supplement: Supplemental data [file Supp_FigS1.pdf]

Supplementary Data

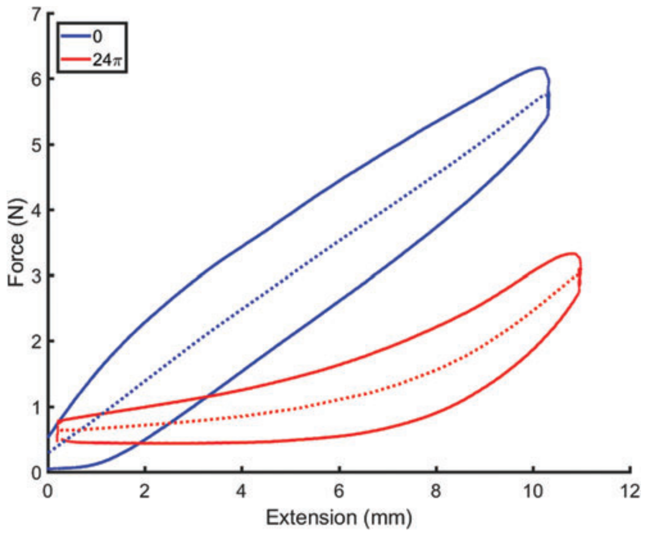

SUPPLEMENTARY FIG. S1. Force-extension behavior of a 15 cm long 3 mm diameter sample of flouroelastomer (FKM standard) rubber cord. *Dotted lines* are averages of lengthening and shortening data. Each *line* shows data for one twist angle, either 0 or 24π.
